# The impact of the COVID-19 pandemic on nurses in Europe: A critical discussion of policy failures and opportunities for future preparedness

**DOI:** 10.1016/j.ijnsa.2021.100032

**Published:** 2021-05-26

**Authors:** Paul De Raeve, Elizabeth Adams, Andreas Xyrichis

**Affiliations:** a European Federation of Nurses Associations, Clos du Parnasse, Brussels, Belgium; b Florence Nightingale Faculty of Nursing, Midwifery and Palliative Care, King's College London, London, UK, 57 Waterloo Road, London, United Kingdom

**Keywords:** COVID-19, Nursing, Workforce, Health policy, Europe, Pandemic, Preparedness

## 1 Background

In the current COVID-19 pandemic climate, ensuring the uninterrupted provision of safe and quality healthcare in Europe, and across the globe, hinges on frontline healthcare professionals working safely to their full scope of practice. Nurses are the single largest, and most trusted, professional group in healthcare, delivering the majority of care 24 h a day, seven days a week and 365 days a year (Rosa et al., 2020). Nurses can be described as the linchpin around which healthcare is organised and delivered; the current pandemic has left little room for doubt on the irreplaceable contribution of nurses in health service delivery. Supporting healthcare professionals is a common priority across many countries of the world, and within the European Union in particular (EC, 2020), but commentators agree that workforce actions have so far been limited (Bourgeault et al., 2020), appearing largely ad hoc and disjointed. Here, we raise attention to key issues surrounding the impact of the COVID-19 pandemic on nurses in Europe through a critical discussion based on two recent pan-European surveys, and resulting policy reports (EFN 2020a, b), commissioned by the European Federation of Nurses’ Associations (EFN). We seek to summarize and amplify key messages concerning the failures and opportunities of pandemic management at European level, as well as highlight key messages to inform future pandemic preparedness in Europe and beyond.

### 1.1 Data sources

Responding to rising concerns from nurses across Europe, the European Federation of Nurses’ Associations initiated two strategic actions to gather intelligence from frontline nurses to inform policy responses at national and European level. First, it commissioned a Europe-wide survey among its Members, consisting of 36 National Nurses Associations with a combined membership of three million nurses. The survey was completed through an online questionnaire over September 2020 and gathered data related to COVID-19 and its impact on nurses across Europe (EFN, 2020a). Second, the Members met online for their bi-annual General Assembly in October 2020 and took the opportunity to share key information on the management of COVID-19 at national level (EFN, 2020b). Data from all the Members participating in the General Assembly were collected through the completion of an online, semi-structured, standardised data collection form, and through online group discussions.

The collected data were analysed quantitatively, using descriptive statistics (percentages, counts), and qualitatively using content analysis, and have been summarised as policy reports for dissemination with the European Institutions (EFN, 2020a,b). Through identifying shared concerns across different countries, the European Federation of Nurses’ Associations presented these reports as resources for healthcare stakeholders, policy makers, clinicians, patients and the public to invite discussion, policy response and exchange of practice; ultimately seeking to strengthen resilience in health systems across Europe during and after the COVID-19 pandemic. Below, we share key messages from both these reports highlighting the variation in impact and response across countries in Europe; this is not to critique or rank countries, but rather to inform future work at European Union and national level, and to foster development of connections to support each other in this joint effort to combat the COVID-19 pandemic.

## 2 The impact of COVID-19 on nurses in europe

The September and October 2020 surveys of the European Federation of Nurses’ Associations achieved a 63% (EFN, 2020a) and 77% (EFN, 2020b) response rate respectively, representing input from National Nurses Associations of 27 countries (Albania, Austria, Belgium, Bulgaria, Cyprus, Czech Republic, Denmark, Estonia, Finland, France, Germany, Greece, Iceland, Ireland, Italy, Latvia, Lithuania, Montenegro, Norway, Poland, Portugal, Romania, Slovenia, Spain, Sweden, Switzerland, and the United Kingdom). Overall, the data submitted by the Members present a distressing picture with regard to nurses and nursing in Europe, as outlined below.

### 2.1 Hospitalisations, nurse infections and deaths

The impact of the pandemic on health service delivery in Europe has been significant, with reported (EFN, 2020b) hospitalisations relating to COVID-19 remaining high across countries (average 38,764; range 210–231,217) with negative consequences for the work and workload of frontline nurses. The direct impact on nurses has also been significant, with high numbers reported of nurses infected with COVID-19 (average 2623; range 49 – 16,000). Moreover, at least 101 nurses have died from COVID-19 across Europe; we note that these numbers should be taken as conservative estimates, based on Members’ intelligence, since official records of nurse infections and deaths are not publicly available in many countries in Europe.

### 2.2 Governmental responses to nurse infections

Data from the Members (EFN, 2020a) showed that exposure to COVID-19 was legally recognised as an occupational injury in most countries. Moreover, Members indicated some availability of government compensation to healthcare workers exposed to or infected by COVID-19 in the workplace; however, this was not universal, with Members in some countries noting that such compensation was lacking or inconsistently applied depending on nurses’ area of practice (e.g. primary or acute care) and employer (e.g. public or private). In some countries, governments and employers offered salary supplements (also known as COVID-19 bonus) to frontline workers in recognition of the risk they accepted, both to themselves and their family members, while providing care to patients with COVID-19.

### 2.3 Availability of testing and protective equipment

Alarmingly, Members reported (EFN, 2020b) inconsistency and uncertainty with regard to steady provision of personal protective equipment as well as COVID-19 testing for nurses, irrespective of setting; nurses employed in care homes in Europe were especially concerned. Only a third (*n* = 8) of Members reported availability of testing for nurses regardless of symptoms or exposure. The capacity of healthcare systems in Europe to respond to the pandemic appears to have hinged on nurses’ flexibility to work irrespective of personal protective equipment and testing availability, accepting to take on excess risk, and work above their usual working hours and in settings outside their areas of expertise.

### 2.4 Psychosocial impact on nurses

Not surprisingly, the psychological impact the COVID-19 pandemic has had on nurses in Europe is substantial, with half of Members who responded (*n* = 13, 48%) being concerned about increasing levels of burnout among nurses, intentions to quit the profession and worsening of the nurse shortage (EFN, 2020b). In addition, nurses among some Members (*n* = 12, 44%) also experienced negative social consequences including violence and stigmatization; for example, some nurses were verbally assaulted, and others asked to leave their rental accommodations by housemates and landlords. Disappointingly, Members reported that psychological and social support for those affected by the pandemic was not readily available at a national level in all countries; rather, in most countries, it appeared to depend on voluntary, charitable or private initiatives, and to vary depending on health setting and employer.

## 3 Discussion

Overall, the evidence from the European Federation of Nurses’ Associations surveys point to an inconsistent response to COVID-19 across countries in Europe potentially leading to nurses in some countries finding themselves at a disadvantage. There appears to be significant disparity among employers both across and within countries in Europe, which serves to add rather than alleviate the challenges that face frontline nurses. Similar concerns have been raised in other continents, such as in Australia, where accurate cross-state data remain difficult to collate reliably (Quigley et al., 2020). Given the bleak picture painted above, Europe-wide initiatives may be needed to protect, support, and encourage nurses to continue their important work safely. To ensure success, such initiatives should be co-developed and co-produced with frontline nurses by involving them and their representatives at the highest levels of national, European and international health policy decision making (Rosa et al., 2020).

The COVID-19 pandemic highlighted the importance of consistent support to frontline nurses when such health emergencies arise, especially considering the central role nurses play in pandemic response. As Rosa et al. (2020, p2) noted, nurses are: “first responders, researchers, community liaisons, intensive care experts, ethics experts; and healthcare coordinators, managers, and mobilizers of resources.” It is therefore important to ensure the contribution of nurses is communicated clearly with policy stakeholders and the European public, which could aid in promoting nursing as a worthwhile and rewarding career option; and help overcome the risk of a worsening nurse shortage predicted by Members of the European Federation of Nurses’ Associations.

In addition, nurses in the European Union would arguably be reassured by the development of a consistent approach across member states about the identification of exposure to COVID-19 as an occupational injury, and provision of compensation as appropriate in instances where nurses and their family members have been significantly adversely affected. Moreover, essential post-viral care should be provided to those nurses infected with COVID-19 especially when they are unable to return to work due to suffering from long-term effects of the virus. Further to the negative physical effects, nurses may also develop long-term psychological effects as a result of their exposure to large doses of death, human suffering, moral injury, and burnout (Blanco-Donoso et al., 2020); necessitating ongoing psychosocial support plans.

Finally, on the education and research front, there should be a coordinated capacity building effort for and led by the nursing workforce to ensure access to vital education and training, including on violence and stigmatization. While psychological risks to nurses have also been found in studies from outside Europe, with some noting high rates (>47%) of depression among nurses caring for COVID-19 patients (e.g. Zheng et al., 2020), social risks to nurses as reported by Members of the European Federation of Nurses’ Associations have hitherto been overlooked. A call to examine the drivers, mechanisms and consequences of COVID-19-related violence and stigmatization should be included within the European Health Research Programme to inform development of evidence-based preventive and support initiatives.

### 3.1 Policy implications

The data seen by the European Federation of Nurses’ Associations suggest that the resilience noted in health systems across Europe to date is partly due to the flexibility, commitment and dedication of frontline nurses. Nurses’ ethos during the COVID-19 pandemic has also been noted in studies completed in North America, where nurses reported a “sense of duty” despite concerns of contracting or spreading infection among their loved ones (Shroeder et al., 2020). It would therefore appear imperative that European Institutions and other policy stakeholders increase their efforts to ensure nurses are adequately protected and supported; this includes protection from physical, psychological as well as social risks. Consequently, the Members of the European Federation of Nurses’ Associations have urged the European Institutions to:

• allocate dedicated funding for the provision of essential protective and care equipment to frontline nurses, irrespective of health setting (community, primary or acute care) or employer (public or private);
• invest in increasing nurse staffing numbers through appropriate recruitment, retention and training initiatives;
• provide psychological, physical, financial and social support to nurses adversely affected by the pandemic; and,
• ensure a safe workplace for nurses through following available legislation for appropriate health and safety measures and positive work environments.

## 4 Conclusions

Through the European Federation of Nurses’ Associations and its Members, three million nurses across the European Union urge health policy stakeholders at all levels to listen to and protect those on the COVID-19 frontline. It is those on the frontline who ultimately act to safeguard health services against the ever-increasing threat of pandemics in Europe. The Members acknowledge that the best time to have started preparing for the current COVID-19 pandemic was back in 2015, following the Ebola crisis in Europe (EFN, 2015); the second-best time is now. Preparing now can only benefit any future response, as well as restore confidence in Europe's health systems more widely. It is vital for the European Institutions and other policy stakeholders to invest in the nursing profession now, to safeguard Europe from further devastating effects of the current and future pandemics.

## Declaration of Competing Interest

None - the authors declare no known conflicts of interest.
